# Practice patterns for eosinophilic esophagitis vary widely among Canadian gastroenterologists: a nationwide survey

**DOI:** 10.1093/jcag/gwae033

**Published:** 2024-10-29

**Authors:** Andrew Fetz, Alexander R Hemy, Hyun Jae Kim, Sarvee Moosavi

**Affiliations:** Division of Gastroenterology, Department of Medicine, University of British Columbia, Vancouver General Hospital, Vancouver, BC V5Z 1M9, Canada; Department of Medicine, University of British Columbia, Vancouver, BC V5Z 1M9, Canada; Division of Gastroenterology, Department of Medicine, University of British Columbia, Vancouver General Hospital, Vancouver, BC V5Z 1M9, Canada; Division of Gastroenterology, Department of Medicine, University of British Columbia, Vancouver General Hospital, Vancouver, BC V5Z 1M9, Canada

**Keywords:** eosinophilic esophagitis, practice patterns, Canadian gastroenterologists

## Abstract

**Introduction:**

Eosinophilic esophagitis (EoE) is a chronic allergic, type 2, immune-mediated condition of the oesophagus, resulting in dysmotility and oesophageal stricturing. This study aims to identify practice variation among Canadian gastroenterologists treating adults with EoE.

**Methods:**

A cross-sectional, web-based survey was distributed to Canadian gastroenterologists through the Canadian Association of Gastroenterology and administrations of Canadian universities.

**Results:**

Seventy gastroenterologists completed the survey, with 59% working in academic practice or research. Overall, 90% of gastroenterologists require histological evidence of EoE to establish a diagnosis of EoE, while 50% require clinical symptoms of oesophageal dysfunction; 39% of gastroenterologists take less than 5 biopsies when assessing for EoE, with variability in biopsy location. Only 51% of respondents took biopsies in every case presenting with acute food bolus. Proton pump inhibitors

were the initial therapy of 70% of gastroenterologists, with 11% using topical steroids. The preferred dietary approach was the 6-food elimination diet in 36%, followed by the 2-food elimination diet in 26%. Overall, 27% of participants did not use histologic improvement and 63% did not use endoscopic improvement to evaluate treatment response. Use of EoE Endoscopic Reference Score (EREFS) is low, with 56% being either unaware of what EREFS is or never using it. Most respondents feel Canadian guidelines would be helpful in their practice.

**Conclusions:**

Eosinophilic esophagitis practice patterns among Canadian gastroenterologists are variable and differ from consensus guidelines. The development of Canadian guidelines and continuing medical education content can be considered to improve the management of EoE in Canada.

## Introduction

Eosinophilic esophagitis (EoE) is a chronic, type 2, immune-mediated allergic condition characterized by infiltration of eosinophils into the oesophageal mucosa and submucosa, leading to inflammation, fibrosis, and oesophageal dysfunction. The incidence of EoE in Canada is estimated at 6.7 per 100 000 inhabitant-years while the prevalence is reported at 33.7 per 100 000 inhabitant-years.^1^ The incidence and prevalence of EoE have increased over the last 5 decades, likely due to changes in diagnostic criteria, increased recognition, increased oesophageal biopsies performed, changes in health care–related early life exposures, and an overall increase in the prevalence of atopic diseases.^1^

Eosinophilic esophagitis develops from a combination of genetic and environmental exposures, resulting in a Th-2 mediated immune response.^2^ Eosinophilic esophagitis is closely associated with atopic disorders, such as asthma and eczema, and an allergic response to food allergens can result in histological evidence of oesophageal eosinophilia.^2^ Genetic alternations in epithelial cell function and gastroesophageal reflux contribute to impaired oesophageal epithelial cell function and increased barrier permeability, which further propagates allergen exposure and inflammation.^2^ Uncontrolled inflammation from eosinophilic infiltration results in oesophageal remodelling, fibrosis, dysmotility, and eventual progression to stricturing.

The presentation and symptoms of EoE vary by age; infants can experience abdominal pain, vomiting, irritability, and feeding problems, whereas adolescents and adults are more likely to present with dysphagia, chest pain, and food impaction.^3^

Diagnosis of EoE by current consensus requires both oesophageal dysfunction and histological findings of ≥15 eosinophils/high power field on oesophageal biopsies.^4^ As of the 2018 AGREE Conference, the exclusion of gastroesophageal reflux disease with a trial of proton pump inhibitors (PPI) has been removed from the diagnostic criteria.^4^ The severity of EoE can be graded using the EoE Endoscopic Reference Score (EREFS), which quantifies endoscopic findings such as oedema, fixed rings, exudates, furrows, and strictures.^5^

Treatment of EoE consists of a combination of dietary, medication, and procedural interventions. Dietary changes include the elemental diet and food elimination diets (FED).^6–8^ The elemental diet is a medically prescribed nutritional product, which is the most restrictive dietary approach to EoE.^9^ The 6FED consists of removal of dairy, wheat or gluten, eggs, soy, nuts, and seafood, while the 4FED allows nuts and seafood.^8^ The milk-elimination diet (MED) eliminates all animal-milk products.^10^ Pharmacotherapy includes PPI, topical corticosteroids (TCs), and, more recently, targeted EoE-specific biologics.^11–15^ Endoscopic intervention with dilation is performed in patients with strictures.^16^

Despite the increasing incidence of EoE, no Canadian guidelines are available to standardize management. This study was conducted to assess practice patterns and variations among Canadian gastroenterologists in the diagnosis, management, and follow-up care of patients with EoE.

## Methods

A cross-sectional, web-based survey was developed to assess practice patterns of Canadian gastroenterologists managing EoE. Only province and specialty of practice were collected as identifiable data. All participants provided consent for study participation.

### Participant recruitment

All adult gastroenterologists practicing in Canada were eligible to participate in the survey. Paediatric gastroenterologists, endoscopists from non-gastroenterology specialties such as surgery, and trainees were not included. Participants were recruited through the Canadian Association of Gastroenterology website/surveys and newsletter, as well as by emails via the administration of Canadian universities. Repeat submissions were not accepted. No further sampling of this population was completed. Sample size calculations were not performed due to a large number of outcomes tested in the survey. The primary study outcome was a descriptive view of the practice patterns among gastroenterologists rather than a comparative analysis. Due to the methods used for survey distribution, it was not possible to determine the survey response rate.

### Survey design and administration

A web-based survey was administered using Google Forms (Google LLC) between May 2023 and June 2024. Survey design prevented submission of incomplete results. Survey pre-testing was not completed.

The survey included a total of 50 questions consisting of multiple-choice questions. Survey sections included participant demographics (age, gender, and time since training), practice setting (province or territory, community or academic, and specialty interest), experience with management of EoE, diagnostic approach, treatment preferences, and follow-up monitoring. Participants were asked to evaluate their knowledge and comfort with treatment for EoE, as well as their practice patterns for diagnosis and follow-up based on the percentage of cases they estimate performing a specific evaluation or intervention. Survey questions are available in Appendix SI.

### Statistical analysis

Descriptive statistics with counts and percentages were completed for each variable and outcome using Microsoft Excel. Comparative analysis between comfort with the management of EoE and diagnostic and monitoring gaps was performed by chi-square analysis using IBM SPSS Statistics (version 29).

Gastroenterologists were considered comfortable managing EoE if they reported being somewhat comfortable or very comfortable by survey response. Gastroenterologists were considered to have gaps in diagnosis if they reported performing biopsy in <50% of patients with acute food bolus, taking <5 biopsies when diagnosing EoE, not taking biopsies from the distal and mid or proximal oesophagus, or not requiring eosinophilic-predominant inflammation on oesophageal biopsy to establish the diagnosis of EoE. Gastroenterologists were considered to have gaps in monitoring if they reported using EREFS less than 50% of the time when assessing EoE or being unfamiliar with EREFS, managed treatment based on improving symptoms rather than repeat endoscopic assessment and biopsies, or not performing regular follow-up endoscopy to monitor patients.

## Results

A total of 70 gastroenterologists completed the survey. Participant demographics are provided in Table 1. Male participants represented 76% of survey respondents. All respondents were adult gastroenterologists. Most gastroenterologists (41, 59%) worked in either academic practice or mainly research, while the remainder worked in urban or rural community practice. Reported specialties and interests included therapeutic endoscopy (19, 27%), GI motility and neurogastroenterology (12, 17%), inflammatory bowel disease (8, 11%), transplant hepatology (5, 7%), non-transplant hepatology (5, 7%), and nutrition (2, 3%); 19 (27%) had no specific specialty or interest.

**Table 1.** Demographics and practice settings of survey respondents.

	Number of participants
Age	
30-39 years	25
40-49 years	27
50-59 years	10
60-69 years	5
Over 70 years	3
Gender	
Male	53
Female	17
Province or territory	
Alberta	9
British Columbia	37
Manitoba	5
Ontario	12
Quebec	3
Saskatchewan	3
Yukon	1
Practice setting	
Academic gastroenterology	40
Community gastroenterology—urban	27
Community gastroenterology—rural	2
Mainly research	1
Time since gastroenterology training	
0-5 years	23
6-14 years	27
15-24 years	12
≥25 years	8
Subspecialty or interest	
None	19
Therapeutic endoscopy	19
GI motility/neurogastroenterology	12
Inflammatory bowel disease	8
Transplant hepatology	5
Non-transplant hepatology	5
Nutrition	2

Most gastroenterologists (90%) reported being somewhat comfortable or very comfortable with managing patients with EoE, and 40% of participants manage at least 20 patients with EoE per year.

### Diagnosis

Diagnostic patterns are reported in Table 2. The 2022 American Society of Gastrointestinal Endoscopy (ASGE) guidelines recommend performing at least 6 oesophageal biopsies from the distal and mid/proximal oesophageal when diagnosing EoE, as well as performing biopsies in patients presenting with food impaction.^16^ The percentage of gastroenterologists who took 4 or less oesophageal biopsies was 39%. Preferred biopsy locations were variable; 11 (16%) biopsied only one location. Most gastroenterologists (56, 80%) biopsy the distal oesophagus, 50 (71%) biopsy the mid oesophagus, and 36 (51%) biopsy the proximal oesophagus. The majority of gastroenterologists (56, 80%) biopsy both the distal and mid or proximal oesophagus, or all 3 locations. Half of respondents (51%) took biopsies in every case presenting with food impaction, while 14% took biopsies less than 25% of the time or only when there were suggestive endoscopic features of EoE.

**Table 2.** Diagnostic approach to eosinophilic esophagitis (EoE).

	Number of participants
How often do you obtain biopsies in a patient presenting with an acute food bolus?	
Every case	36
Over 50% of cases but not always	18
Occasionally (more than 25% but less than 50% of cases)	6
Rarely (less than 25% of cases)	7
Only if endoscopic findings are suggestive of EoE	2
Never	1
Biopsy locations*	
Distal oesophagus	56
Mid oesophagus	50
Proximal oesophagus	36
Number of biopsies	
1-4	27
5-8	33
9-12	9
More than 12	1
Targeted vs random biopsies	
Random	26
Targeted	14
Both	30

*More than one response allowed.

**Figure 1.**
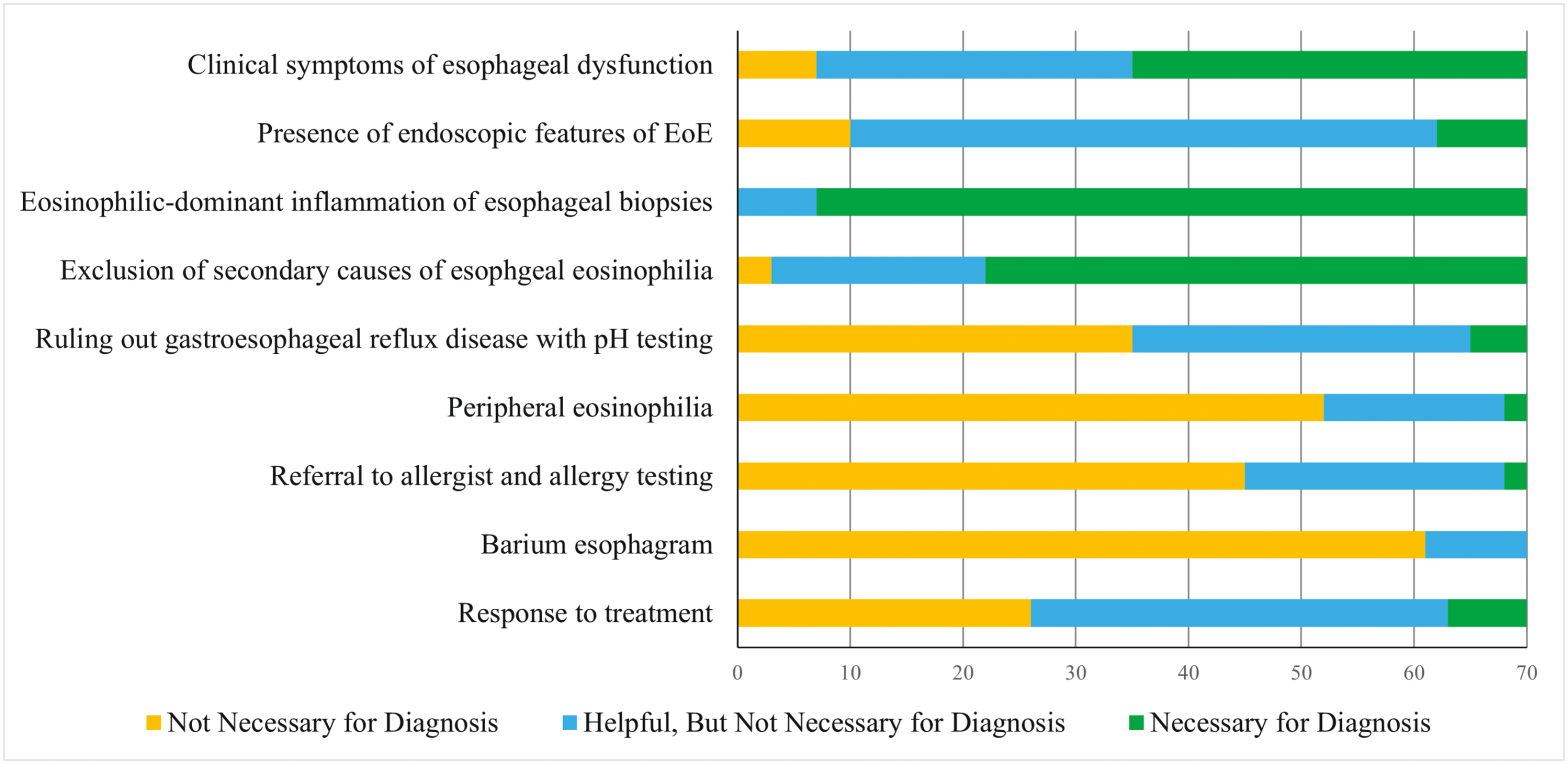
Factors necessary for eosinophilic esophagitis diagnosis reported by respondents.

Diagnostic criteria are displayed in Figure 1. The diagnosis of EoE requires symptoms of oesophageal dysfunction, oesophageal eosinophilia >15 eosinophils/high power field on biopsy, and evaluation of non-EoE disorders that could contribute to oesophageal eosinophilia.^4^ Nearly all gastroenterologists (90%) required eosinophil-predominant inflammation on oesophageal biopsy for the diagnosis of EoE. Half of gastroenterologists do not require clinical features of oesophageal dysfunction to diagnose EoE. About two-thirds (69%) reported that exclusion of secondary causes of oesophageal eosinophilia was required for diagnosis. Diagnostic gaps were found in 63.5% of gastroenterologists who reported they were comfortable with management of EoE, compared to 42.9% of gastroenterologists who did not report they were comfortable with management of EoE. There was no association between diagnostic gaps and reported comfort with management of EoE (*Χ*2 *n* = 70) = 1.132, *P* = .287.

### Treatment

Treatment practice patterns are provided in Table 3. The 2020 American Gastroenterology Association (AGA) guidelines recommend proton pump inhibitors (PPI), TC, elemental diet, 6FED, and allergy testing–based elimination diets as treatments for EoE.^11^ Monotherapy with PPIs was the preferred initial pharmacotherapy by most gastroenterologists (70%). Thirteen participants (19%) reported using a combination approach as their initial pharmacotherapy, while the remainder used TC.

**Table 3.** Treatment of eosinophilic esophagitis (EoE) with proton pump inhibitors, topical corticosteroids, elimination diets, and biologics.

	Number of participants
Preferred primary treatment for EoE	
Proton pump inhibitors	49
Topical corticosteroids	8
Multiple therapies	13
Recommended elimination diet	
6FED	25
4FED	11
2FED	18
Milk-only elimination diet	10
Allergy test–directed diet	4
Elemental diet	2
Familiarity with data for biologic therapies	
Very familiar	12
Somewhat familiar	40
Not familiar	3
Aware these existed	13
Unaware these options existed	2
Familiarity with prescribing biologics	
Very comfortable	10
Somewhat comfortable	22
Not very comfortable	23
Not at all comfortable	13
How often do you work towards discontinuation of topical corticosteroids?	
All cases and monitor for symptom recurrence	23
In most cases (greater than 50%)	25
Only for patient preference after initial remission is achieved	18
Never, my patients stay on topical steroids indefinitely	4

The 6FED was the preferred diet for 25 gastroenterologists (36%). The 4FED was preferred by 11 gastroenterologists (16%), while the 2FED was preferred by 18 (26%) and the MED was preferred by 10 (14%). Few gastroenterologists preferred the elemental diet (2, 3%) or allergy test–directed diet (4, 6%).

Most gastroenterologists (52, 74%) are very familiar or somewhat familiar with EoE-indicated biologic therapies, while only 32 (46%) feel comfortable with prescribing these treatments for their patients.

When using TCs, two-thirds of gastroenterologists (69%) report they work towards discontinuation, whereas the AGA recommends continuation of TC over discontinuation of treatment.^11^ Additionally, most gastroenterologists (83%) will counsel their patients on treatment options and the expected disease course of EoE. Referral to an allergist after diagnosis is low, with most respondents (66%) referring less than half of their patients. Five respondents only refer patients with a history of prior allergies, while 2 respondents refer to help with elimination diets.

### Follow-up and monitoring

The ASGE recommends using endoscopy and biopsy sampling rather than symptomatic improvement to assess EoE activity before and after any change in treatment.^16^ Most respondents (53, 76%) use symptomatic improvement to evaluate treatment efficacy, while 17 (24%) use improving symptoms alone as a measure of treatment success, 73% use histologic improvement, and 37% use improvement in endoscopic optical evaluation. Despite this, most respondents (71%) reported that symptoms of EoE do not accurately predict disease activity. Screening for adaptive behaviours, such as increased chewing or texture modification, is inconsistent, with 37% of respondents assessing these behaviours less than half the time or not at all.

More than half of gastroenterologists (51%) re-evaluate all patients with esophagogastroduodenoscopy (EGD) to assess treatment efficacy; 23 respondents (33%) only perform EGD for those who remain symptomatic and 8 respondents (11%) perform EGD in those who require dilation. When repeat EGD is performed, 8 (11%) reassess within 3 months, 23 (33%) reassess within 3-5.9 months, and 23 (33%) reassess between 6 and 11.9 months. Seven gastroenterologists (10%) only re-evaluate based on symptom recurrence rather than repeat EGD to assess EOE activity. EREFS utilization is low, with 41 (59%) reporting they never use EREFS or are unaware of what EREFS is.

Gaps in follow-up monitoring were found in 82.5% of gastroenterologists who reported comfort with managing EoE, and 85.7% of gastroenterologists who did not report comfort with management of EoE. There was no significant association between reported comfort with management of EoE and gaps in monitoring (*Χ*2 *n* = 70) = 0.045, *P* = .833.

### Guidelines

The preferred guidelines for assessment and management of EoE were the 2020 AGA guidelines and the 2022 ASGE guidelines (93%). Most participants (86%) reported that they would consider Canadian EoE guidelines helpful for the workup and management of EoE in their practice.

## Discussion

Eosinophilic esophagitis practice patterns are variable among Canadian gastroenterologists, differing from American EoE guideline recommendations in diagnosis, treatment, and disease monitoring.

Despite an increasing incidence and recognition of EoE, patients often experience a delayed diagnosis, with a median time to diagnosis of 4 years, and nearly one-third of patients being delayed for more than 10 years.^17^ The ASGE recommends taking at least 6 biopsies and taking biopsies from multiple locations including both the distal and mid/proximal oesophagus due to the patchy distribution of EoE.^16^ Additionally, the ASGE recommends taking biopsies regardless of a normal endoscopic appearance due to the low sensitivity of endoscopy for diagnosis of EoE and potential for missed diagnosis by less experienced endoscopists.^16^ In our study, one-third of those taking biopsies take fewer than the recommended number of biopsies, while 20% do not take biopsies from all recommended locations.

The ASGE also recommends taking biopsies on initial endoscopy for patients presenting with food impaction.^16^ Only half of gastroenterologists obtain routine biopsies in patients with food impaction despite a high prevalence of EoE.^18^ These results suggest that testing and recognition of EoE remains suboptimal. Prompt diagnosis of EoE has important implications on patient outcomes as delays in diagnosis are associated with disease progression and development of oesophageal strictures.^19^

Eosinophilic esophagitis requires ongoing treatment as a chronic condition, with multiple dietary, pharmacological, and endoscopic interventions available. Dietary elimination strategies are effective in achieving histological and clinical response.^6^ Among Canadian gastroenterologists, there is significant variability in the preferred dietary approach for management of EoE. The elemental diet is the most restrictive approach, consisting of a medically prescribed liquid diet that achieves histological remission in 93.6% of patients, and is a potential treatment option for refractory EoE.^20,21^ Although efficacious, the elemental diet was previously limited by poor adherence in 38% of patients, as well as weight loss, high costs, and histologic relapse after liberalization of dietary restrictions.^7^ More recently, the use of new flavoured elemental diet products has been associated with near complete adherence in clinical trials.^9^ None of the FEDs have demonstrated superiority in meta-analysis, although the data is mostly retrospective and low quality.^6^ FEDs can be difficult to maintain long-term, and are associated with increased costs and difficulty with acquiring food supplies.^22^ As such, strategies such as MED or a step-up approach can be used to minimize the burden on patients and potentially improve adherence.^10,23^ Despite MED being non-inferior to 6FED, only 14% of gastroenterologists surveyed recommend MED as their initial dietary approach.^10^ Based on these findings, variability is expected and shared decision-making between physicians and patients may be reasonable when selecting dietary interventions.

Initial pharmacotherapy for EoE includes PPIs and/or TCs.^11^ PPIs are effective at achieving histologic remission and improving endoscopic findings by EREFS, however, the effect on symptomatic improvement is not proven.^12^ Based on observational studies, 41.7% of patients treated with PPI will achieve histologic remission.^20^ Canadian gastroenterologists preferentially prescribe PPIs as their initial therapy for EoE, with only 11% using TCs as their first treatment. PPIs remain a reasonable first-line treatment due to their ease of use and favourable safety profile, particularly after removal of PPI non-responders from the EoE diagnostic criteria.^4,11^

Topical corticosteroids (TCs), such as budesonide slurry and swallowed fluticasone, are effective treatments to improve symptoms, achieve histological remission and endoscopic improvement by EREFS in EoE.^12^ Budesonide orodispersible tablets (BOT), such as Jorveza, are the first oral TC formulation developed and approved in Canada for the treatment of EoE, in contrast to the off-label use of swallowed asthma medications. BOT results in histological remission in 93.2% of patients after 6 weeks of treatment, and clinico-histologic remission in 84.7% after 12 weeks of treatment.^13^ Despite these findings, BOT does not currently have Food and Drug Administration approval in the United States, limiting American guideline recommendations.

The first biologic therapy approved for EoE is dupilumab, which was approved for use in Canada in May 2023, but formal Canadian guidelines regarding its use are lacking. Dupilumab is a human monoclonal antibody that blocks the receptors for interleukin-4 and interleukin-13, inhibiting signalling pathways in type 2 inflammation. Dupilumab is effective for patients with EoE who have not responded to PPI therapy, with histologic remission achieved in 60% of patients at week 24.^14^

There are other biologics that are currently in clinical trials, including benralizumab, an anti–interleukin-5 receptor alpha monoclonal antibody, previously used to treat eosinophilic asthma. In a recent study, benralizumab was shown to induce rapid, near-complete eosinophil depletion; however, dysphagia did not differ between the treatment and control groups.^24^ Mepolizumab and reslizumab, targeted treatments against interleukin-5, reduce oesophageal eosinophilia compared to placebo but do not significantly improve symptoms. Similarly, lirentelimab, a monoclonal antibody against siglec-8, also demonstrated histological response, although there was no significant improvement in dysphagia compared to placebo. Cendakimab, a monoclonal antibody against interleukin-13, has shown improvement in oesophageal eosinophilia and endoscopic findings of EoE by EREFS, as well as a trend towards symptomatic improvement, and remains under investigation in phase 3 clinical trials.^15^

Additional therapies, such as montelukast, cromolyn sodium, and immunomodulators, remain under investigation and are not currently recommended by the AGA to treat EoE outside of clinical trials.^11^ Given the recency in availability of biologics for EoE in Canada, it is not surprising that their use by Canadian gastroenterologists remains limited, with only 17% of respondents being very comfortable with starting biologic therapies to treat EoE.

When managing EoE, two-thirds of gastroenterologists do not routinely involve allergists in the care of their patients. The ASGE does not provide a recommendation regarding the role of allergists, whereas the AGA recommends allergy testing–based elimination diet over no treatment.^11,16^ Given that only 6% of gastroenterologist report using an allergy test–directed diet, it is not surprising that the studied population has a low rate of referral to allergists, and further guideline-based recommendations would be needed to clarify the role of allergist in the management of adult patients with EoE.

Monitoring of EoE can be challenging, as symptoms do not correlate well with histological findings and clinicians should avoid using symptomatic improvement alone as a treatment endpoint.^25^ This is particularly troublesome when oesophageal dilation is used in symptomatic management, as patients may experience temporary relief of symptoms without histological response.^25^ Patients with EoE can also develop adaptive eating behaviours, such as prolonged meal times, excessive chewing, increased water intake, and certain food texture avoidance to adapt to ongoing dysphagia, which can falsely reassure providers.^26^ Despite this, gastroenterologists reported not routinely screening for adaptive eating behaviours. Our results suggest that objective measures of EoE activity are under-utilized in Canadian practice, with over half of Canadian gastroenterologists not knowing or utilizing the EREFS score to objectively monitor endoscopic findings. Additionally, over one-quarter of gastroenterologists do not monitor ongoing histological activity with biopsies for patients with confirmed EoE.

The ASGE recommends repeating endoscopic assessment with biopsies 4 weeks after changing FED and 8-12 weeks after changing pharmacotherapy to establish an objective response to therapy; monitoring improvement of symptoms or endoscopic appearance alone is not recommended.^16^ Patients with EoE who undergo more frequent follow-up with an interval of less than 18 months are more likely to detect histologic relapse early and are less likely to develop strictures, supporting a need for regular monitoring and endoscopic assessment.^27^

Eosinophilic esophagitis should be approached as a chronic condition requiring maintenance therapy to sustain endoscopic and histological remission. The use of endoscopic and histologic response as treatment outcomes appears to have similarities to those recommended in the STRIDE-II guidelines for the management of Crohn’s disease, although further evidence is needed to guide long-term treatment outcomes in EoE and draw such parallel comparisons.^28^ The best approach to maintenance therapy for EoE remains an area of ongoing research, with many trials to date focusing on short-term outcomes.^11^ Dietary interventions are effective at maintaining histological remission, with up to 60% of patients on a FED achieving remission and 72% of those on an elemental diet, although these interventions are limited by non-adherence to treatment and relapse after re-introduction of triggering foods.^6,7,29,30^

Topical corticosteroids (TCs) are more likely to achieve sustained remission compared to placebo, with 75% of responders to BOT maintaining histological remission over 48 weeks.^31^ The current AGA guidelines suggest the continuation of TCs due to the potential for long-term progression from inflammation to oesophageal strictures in a proportion of EoE patients with untreated disease.^11^ Interestingly, 69% of respondents in our survey routinely work towards discontinuation of TCs. As maintenance therapy, TCs are safe, with only mild adverse events such as candidiasis and mildly decreased serum morning cortisol, and no increase in serious adverse events.^31^ Discontinuing maintenance therapy can be detrimental for patients, as the development of fibrostenotic changes and strictures may result in persistent symptoms despite histological remission.^11^

Similarly, most patients who achieve histologic remission with PPI therapy will maintain remission during follow-up with maintenance treatment, with 40% losing histologic response over an average of 3.6 years.^32^ Loss of response is more common in younger patients and those requiring baseline oesophageal dilation.^32^

This study has several strengths, supporting the dire need for Canadian EoE guidelines and future educational contents that would further improve the quality of EoE patients’ care. To date, this is the largest survey completed assessing practice patterns in EoE amongst Canadian gastroenterologists. The study was performed at a national level and gathered perspectives from gastroenterologists with a range of years of experience, sub-specialty backgrounds, and practice settings. The study attempted to maximize the participation of gastroenterologists by providing survey access through both university administrations and the Canadian Association of Gastroenterology. The study also has important implications, as it highlights significant deviation from guideline-directed management of EoE among gastroenterologists across Canada, with a significant percentage of gastroenterologists having knowledge gaps in diagnosis and follow-up monitoring of EoE despite a high reported comfort level among participants with managing the condition.

The study has some limitations: the study includes the use of self-reported practice patterns, which may vary from the actual clinical practice audits. In addition, those who responded to the survey may be gastroenterologists with more knowledge or interest in EoE, although, as demonstrated in Table 1, the study did include gastroenterologists from various GI sub-specialties; therefore, this is less likely a significant concern to cause selection bias. Due to the distribution method of the survey, it was not possible to determine a response rate among participants who had received a survey request. Non-participation in the study may have occurred due to lack of time or interest in participation, participants not monitoring emails of the CAG portal, and language barriers in French-speaking gastroenterologists. Our study did include more academic gastroenterologists; however, EoE is commonly encountered in both community and rural practices as well. Finally, the study was limited to adult gastroenterologists to focus on specialists most likely to be involved in the care of adult EoE patients. We did not include non-gastroenterologist endoscopists in this study, including thoracic or general surgeons who may also perform upper endoscopy, yet may be less familiar or confident in diagnosing or managing eosinophilic esophagitis.

In conclusion, this study highlights heterogeneity in clinical practice patterns amongst Canadian gastroenterologists in the diagnosis, treatment, and monitoring of EoE. Like other chronic inflammatory conditions, such as inflammatory bowel disease, prompt recognition and management with an emphasis on maintaining clinical, endoscopic, and ideally histologic remission seem to be essential in optimizing clinical outcomes in EoE and reducing the risk of long-term sequelae, such as fibrostenotic oesophageal remodeling and recurrent food impaction. These findings highlight the need for the development of further training materials and national guidelines to standardize the diagnosis and management of EoE by Canadian gastroenterologists and non-gastroenterologist endoscopists.

## Supplementary material

Supplementary material is available at *Journal of the Canadian Association of Gastroenterology* online.

gwae033_suppl_Supplementary_Materials

## Data Availability

The data underlying this article will be shared on reasonable request to the corresponding author.

## References

[1] Hahn JW , Lee K, Shin JI, et al Global incidence and prevalence of eosinophilic esophagitis, 1976–2022: a systematic review and meta-analysis. Clin Gastroenterol Hepatol. 2023;21(13):3270–3284.e77. https://doi.org/10.1016/j.cgh.2023.06.005 37331411

[2] O’Shea KM , Aceves SS, Dellon ES, et al Pathophysiology of eosinophilic esophagitis. Gastroenterology. 2018;154(2):333–345. https://doi.org/10.1053/j.gastro.2017.06.065 28757265 PMC5787048

[3] Straumann A , Aceves SS, Blanchard C, et al Pediatric and adult eosinophilic esophagitis: similarities and differences. Allergy. 2012;67(4):477–490. https://doi.org/10.1111/j.1398-9995.2012.02787.x 22313241

[4] Dellon ES , Liacouras CA, Molina-Infante J, et al Updated International Consensus Diagnostic Criteria for Eosinophilic Esophagitis: Proceedings of the AGREE Conference. Gastroenterology. 2018;155(4):1022–1033.e10. https://doi.org/10.1053/j.gastro.2018.07.009 30009819 PMC6174113

[5] Hirano I , Moy N, Heckman MG, Thomas CS, Gonsalves N, Achem SR. Endoscopic assessment of the oesophageal features of eosinophilic oesophagitis: validation of a novel classification and grading system. Gut. 2013;62(4):489–495. https://doi.org/10.1136/gutjnl-2011-301817 22619364

[6] Mayerhofer C , Kavallar AM, Aldrian D, Lindner AK, Müller T, Vogel GF. Efficacy of elimination diets in eosinophilic esophagitis: a systematic review and meta-analysis. Clin Gastroenterol Hepatol. 2023;21(9):2197–2210.e3. https://doi.org/10.1016/j.cgh.2023.01.019 36731591

[7] Peterson KA , Byrne KR, Vinson LA, et al Elemental diet induces histologic response in adult eosinophilic esophagitis. Am J Gastroenterol. 2013;108(5):759–766. https://doi.org/10.1038/ajg.2012.468 23381017

[8] Kagalwalla AF , Sentongo TA, Ritz S, et al Effect of six-food elimination diet on clinical and histologic outcomes in eosinophilic esophagitis. Clin Gastroenterol Hepatol. 2006;4(9):1097–1102. https://doi.org/10.1016/j.cgh.2006.05.026 16860614

[9] Warners MJ , Vlieg-Boerstra BJ, Verheij J, et al Elemental diet decreases inflammation and improves symptoms in adult eosinophilic oesophagitis patients. Aliment Pharmacol Ther. 2017;45(6):777–787. https://doi.org/10.1111/apt.13953 28112427 PMC5324627

[10] Kliewer KL , Gonsalves N, Dellon ES, et al One-food versus six-food elimination diet therapy for the treatment of eosinophilic oesophagitis: a multicentre, randomised, open-label trial. Lancet Gastroenterol Hepatol. 2023;8(5):408–421. https://doi.org/10.1016/S2468-1253(23)00012-2 36863390 PMC10102869

[11] Hirano I , Chan ES, Rank MA, et al; AGA Institute Clinical Guidelines Committee. AGA Institute and the Joint Task Force on Allergy-Immunology Practice Parameters Clinical Guidelines for the Management of Eosinophilic Esophagitis. Gastroenterology. 2020;158(6):1776–1786. https://doi.org/10.1053/j.gastro.2020.02.038 32359562 PMC9473154

[12] Visaggi P , Barberio B, Del Corso G, et al Comparison of drugs for active eosinophilic oesophagitis: systematic review and network meta-analysis. Gut. 2023;72(11):2019–2030. https://doi.org/10.1136/gutjnl-2023-329873 37491157

[13] Lucendo AJ , Miehlke S, Schlag C, et al; International EOS-1 Study Group. Efficacy of budesonide orodispersible tablets as induction therapy for eosinophilic esophagitis in a randomized placebo-controlled trial. Gastroenterology. 2019;157(1):74–86.e15. https://doi.org/10.1053/j.gastro.2019.03.025 30922997

[14] Dellon ES , Rothenberg ME, Collins MH, et al Dupilumab in adults and adolescents with eosinophilic esophagitis. N Engl J Med. 2022;387(25):2317–2330. https://doi.org/10.1056/NEJMoa2205982 36546624

[15] Dellon ES , Spergel JM. Biologics in eosinophilic gastrointestinal diseases. Ann Allergy Asthma Immunol. 2023;130(1):21–27. https://doi.org/10.1016/j.anai.2022.06.015 35738437 PMC10191215

[16] Aceves SS , Alexander JA, Baron TH, et al Endoscopic approach to eosinophilic esophagitis: American Society for Gastrointestinal Endoscopy Consensus Conference. Gastrointest Endosc. 2022;96(4):576–592.e1. https://doi.org/10.1016/j.gie.2022.05.013 35965102

[17] Murray FR , Kreienbuehl AS, Greuter T, et al Diagnostic delay in patients with eosinophilic esophagitis has not changed since the first description 30 years ago: diagnostic delay in eosinophilic esophagitis. Am J Gastroenterol. 2022;117(11):1772–1779. https://doi.org/10.14309/ajg.0000000000001950 35971224

[18] Kerlin P , Jones D, Remedios M, Campbell C. Prevalence of eosinophilic esophagitis in adults with food bolus obstruction of the esophagus. J Clin Gastroenterol. 2007;41(4):356–361. https://doi.org/10.1097/01.mcg.0000225590.08825.77 17413601

[19] Schoepfer AM , Safroneeva E, Bussmann C, et al Delay in diagnosis of eosinophilic esophagitis increases risk for stricture formation in a time-dependent manner. Gastroenterology. 2013;145(6):1230–1236. https://doi.org/10.1053/j.gastro.2013.08.015 23954315

[20] Rank MA , Sharaf RN, Furuta GT, et al Technical review on the management of eosinophilic esophagitis. Ann Allergy Asthma Immunol. 2021;124(5):424–440. https://doi.org/10.1016/j.anai.2020.03.021 PMC8171057 32336463

[21] Strauss AL , Falk GW. Refractory eosinophilic esophagitis: what to do when the patient has not responded to proton pump inhibitors, steroids and diet. Curr Opin Gastroenterol. 2022;38(4):395–401. https://doi.org/10.1097/MOG.0000000000000842 35762699 PMC9552275

[22] Sheedy K , Patel N, Porter J, Silva H. Cost and accessibility of empiric food elimination diets for treatment of eosinophilic oesophagitis. Nutr Diet. 2022;79(2):238–246. https://doi.org/10.1111/1747-0080.12717 34927796

[23] Molina-Infante J , Arias A, Alcedo J, et al Step-up empiric elimination diet for pediatric and adult eosinophilic esophagitis: the 2-4-6 study. J Allergy Clin Immunol. 2018;141(4):1365–1372. https://doi.org/10.1016/j.jaci.2017.08.038 29074457

[24] Peterson KA , Brooks L, Sc M, et al Eosinophil depletion with benralizumab for eosinophilic esophagitis. N Eng J Med. 2024;390(24):2252–2263. https://doi.org/10.1056/NEJMoa2313318 38924732

[25] Chang JW , Yeow RY, Waljee AK, Rubenstein JH. Systematic review and meta-regressions: management of eosinophilic esophagitis requires histologic assessment. Dis Esophagus. 2018;31(8):1–9. https://doi.org/10.1093/dote/doy049 PMC9118332 29893826

[26] Alexander R , Alexander JA, Ravi K, et al Measurement of observed eating behaviors in patients with active and inactive eosinophilic esophagitis. Clin Gastroenterol Hepatol. 2019;17(11):2371–2373. https://doi.org/10.1016/j.cgh.2018.12.011 30557737

[27] Bon L , Safroneeva E, Bussmann C, et al Close follow-up is associated with fewer stricture formation and results in earlier detection of histological relapse in the long-term management of eosinophilic esophagitis. United Eur Gastroenterol J. 2022;10(3):308–318. https://doi.org/10.1002/ueg2.12216 PMC9004232 35384368

[28] Turner D , Ricciuto A, Lewis A, et al; International Organization for the Study of IBD. STRIDE-II: An Update on the Selecting Therapeutic Targets in Inflammatory Bowel Disease (STRIDE) Initiative of the International Organization for the Study of IBD (IOIBD): determining therapeutic goals for treat-to-target strategies in IBD. Gastroenterology. 2021;160(5):1570–1583. https://doi.org/10.1053/j.gastro.2020.12.031 33359090

[29] Reed CC , Fan C, Koutlas NT, Shaheen NJ, Dellon ES. Food elimination diets are effective for long-term treatment of adults with eosinophilic oesophagitis. Aliment Pharmacol Ther. 2017;46(9):836–844. https://doi.org/10.1111/apt.14290 28877359 PMC5659358

[30] Gonsalves N , Yang GY, Doerfler B, Ritz S, Ditto AM, Hirano I. Elimination diet effectively treats eosinophilic esophagitis in adults; food reintroduction identifies causative factors. Gastroenterology. 2012;142(7):1451–9.e1; quiz e14. https://doi.org/10.1053/j.gastro.2012.03.001 22391333

[31] Straumann A , Lucendo AJ, Miehlke S, et al; International EOS-2 Study Group. Budesonide orodispersible tablets maintain remission in a randomized, placebo-controlled trial of patients with eosinophilic esophagitis. Gastroenterology. 2020;159(5):1672–1685.e5. https://doi.org/10.1053/j.gastro.2020.07.039 32721437

[32] Thakkar KP , Fowler M, Keene S, Iuga A, Dellon ES. Long-term efficacy of proton pump inhibitors as a treatment modality for eosinophilic esophagitis. Dig Liver Dis. 2022;54(9):1179–1185. https://doi.org/10.1016/j.dld.2022.03.006 35410852 PMC9427674

