# Supplementary material for: Practice patterns for eosinophilic esophagitis vary widely among Canadian gastroenterologists: a nationwide survey
Source: J Can Assoc Gastroenterol. 2024 Oct 29;8(1):13–20. doi: 10.1093/jcag/gwae033 (PMC11788507; doi:10.1093/jcag/gwae033)
Supplement: gwae033_suppl_Supplementary_Materials [file gwae033_suppl_supplementary_materials.zip › Appendix.docx]

Appendix I:

1. What is your current age?
   1. 25 – 29 years
   2. 30 – 39 years
   3. 40 – 49 years
   4. 50 – 59 years
   5. 69 – 69 years
   6. Over 70
2. What is your gender?
   1. Male
   2. Female
   3. Prefer not to disclose
3. Which Province or Territory is your practice located?
   1. AB
   2. BC
   3. MB
   4. NB
   5. NL
   6. NS
   7. NT
   8. NU
   9. ON
   10. PE
   11. QC
   12. SK
   13. YT
4. How would you describe your clinical practice?
   1. Academic gastroenterology
   2. Community gastroenterology – urban
   3. Community gastroenterology – rural (population less than 30,000)
   4. Mainly research, < 50% clinical
5. How many years ago did you complete your core gastroenterology training?
   1. 0 – 5 years
   2. 6 – 14 years
   3. 15 – 24 years
   4. ≥ 25 years
6. Do you have a subspecialty/interest within gastroenterology?
   1. No subspecialty
   2. Therapeutic endoscopy
   3. GI Motility/Neurogastroenterology
   4. Nutrition
   5. IBD
   6. Transplant hepatology
   7. Non-transplant hepatology
7. Approximately how many patients with EoE do you manage annually in your clinical practice?
   1. 1 – 5
   2. 6 – 19
   3. 20 – 50
   4. > 50
8. What groups best describe your EoE practice population?
   1. Adults only
   2. Children only
   3. Mixture of adults and children
9. How comfortable do you feel managing EoE?
   1. Very Comfortable
   2. Somewhat Comfortable
   3. Neutral
   4. Somewhat Uncomfortable
   5. Very Uncomfortable
10. How often do you obtain biopsies in a patient presenting with an acute food bolus?
    1. Every case
    2. Over 50% of cases but not always
    3. Occasionally (more than 25% but less than 50% of cases)
    4. Rarely (less than 25% of cases)
    5. Never
11. What is your approach to initial endoscopic biopsy techniques in suspected EoE?
    1. Location (multi-select)
       1. Distal esophagus
       2. Mid esophagus
       3. Proximal esophagus
    2. Total number of biopsies taken
       1. 1 – 4
       2. 5 – 8
       3. 9 – 12
    3. Targeted versus random (multi-select)
       1. Target biopsy towards the endoscopic abnormalities seen
       2. Random
12. Which of the following do you feel are necessary to make a diagnosis of EoE?
    1. Clinical symptoms of esophageal dysfunction
       1. Necessary for diagnosis
       2. Helpful, but not necessary for diagnosis
       3. Not necessary for diagnosis
    2. Presence of endoscopic features of EoE
       1. Necessary for diagnosis
       2. Helpful, but not necessary for diagnosis
       3. Not necessary for diagnosis
    3. Eosinophilic-predominant inflammation on esophageal biopsy
       1. Necessary for diagnosis
       2. Helpful, but not necessary for diagnosis
       3. Not necessary for diagnosis
    4. Exclusion of secondary causes of esophageal eosinophilia
       1. Necessary for diagnosis
       2. Helpful, but not necessary for diagnosis
       3. Not necessary for diagnosis
    5. Ruling out gastroesophageal reflux disease (GERD) with pH testing
       1. Necessary for diagnosis
       2. Helpful, but not necessary for diagnosis
       3. Not necessary for diagnosis
    6. Peripheral eosinophilia
       1. Necessary for diagnosis
       2. Helpful, but not necessary for diagnosis
       3. Not necessary for diagnosis
    7. Referral to an allergist (e.g. skin allergy testing, atopy patch, or IgE level)
       1. Necessary for diagnosis
       2. Helpful, but not necessary for diagnosis
       3. Not necessary for diagnosis
    8. Barium esophagram
       1. Necessary for diagnosis
       2. Helpful, but not necessary for diagnosis
       3. Not necessary for diagnosis
    9. Response to treatment
       1. Necessary for diagnosis
       2. Helpful, but not necessary for diagnosis
       3. Not necessary for diagnosis
13. When evaluating a patient with EoE, do you use the EREFS score?
    1. Always
    2. In most cases (Over 50%)
    3. Occasionally (Less than 50% or only if they report worsening symptoms)
    4. Never
    5. Unaware of what EREFS is
14. What is your preferred primary treatment?
    1. PPIs
    2. Topical steroids (e.g., swallowed fluticasone, budesonide slurry)
    3. Oral bio-dispersible budesonide (Jorveza)
    4. Leukotriene inhibitors (e.g., montelukast)
    5. Multiple modalities at once (e.g., PPI and dietary elimination, or topical steroids + dietary elimination)
15. If a patient was interested in elimination diet, which dietary approach would you recommend first?
    1. 6FED
    2. 4FED
    3. 2FED
    4. Milk-only elimination
    5. Elemental diet
    6. Allergy test directed diet
16. How familiar are you with the data surrounding biologic therapies for EoE (e.g., anti-IL-5 or anti-IL13/4) ant type II inflammation, and would you feel comfortable initiating this therapy (multi-select)
    1. Very familiar
    2. Somewhat familiar
    3. Aware these existed but not familiar
    4. Not familiar/Unaware these existed
    5. Very comfortable initiating
    6. Somewhat comfortable initiating
    7. Not very comfortable initiating
    8. Not comfortable initiating
17. How do you evaluate for treatment effectiveness (multi-select)
    1. Based on improving patient symptoms
    2. Based on improving endoscopic appearance on repeat EGD
    3. Based on improving histologic activity on repeat biopsies
18. What symptoms do you evaluate on a follow-up visit?
    1. Dysphagia
       1. Always
       2. More than 50%
       3. Less than 50%
       4. Never
    2. Odynophagia
       1. Always
       2. More than 50%
       3. Less than 50%
       4. Never
    3. Chest or abdominal pain
       1. Always
       2. More than 50%
       3. Less than 50%
       4. Never
    4. Sensation of food bolus obstruction
       1. Always
       2. More than 50%
       3. Less than 50%
       4. Never
    5. Skin rashes
       1. Always
       2. More than 50%
       3. Less than 50%
       4. Never
    6. Heart burn
       1. Always
       2. More than 50%
       3. Less than 50%
       4. Never
    7. Regurgitation
       1. Always
       2. More than 50%
       3. Less than 50%
       4. Never
    8. Nausea or vomiting
       1. Always
       2. More than 50%
       3. Less than 50%
       4. Never
    9. Excessive chewing
       1. Always
       2. More than 50%
       3. Less than 50%
       4. Never
    10. Texture modification
        1. Always
        2. More than 50%
        3. Less than 50%
        4. Never
    11. Food or texture avoidance/aversion
        1. Always
        2. More than 50%
        3. Less than 50%
        4. Never
    12. Weight loss
        1. Always
        2. More than 50%
        3. Less than 50%
        4. Never
19. If a patient was started on topical steroid therapies, how often do you work towards discontinuation?
    1. All cases and monitor for symptom recurrence
    2. In most cases (greater than 50%)
    3. Only for patient preference after initial remission is achieved
    4. Never, my patients stay on topical steroids indefinitely
20. If you do discontinue topical steroids therapy, please outline when you decide to stop (e.g., duration of therapy, symptom improvement, whether you stop it after repeating EGD with esophageal biopsies, patient’s request, etc.). If you do not discontinue topical steroid therapy, please write N/A. (Written answer)
21. How well do symptoms predict disease activity in EoE?
    1. Symptoms correlate accurately with disease activity
    2. Symptoms do not correlate well with disease activity
22. How often do you provide patients with counselling on treatment options (including medications, diet elimination, and dilation) and expected disease course (including symptom outcomes and need for future endoscopy) during an initial visit?
    1. Always, during 100% of initial visits
    2. Most of the time, more than half
    3. About half the time
    4. Rarely, less than quarter of the time
    5. Never, 0% initial visits
23. How often do you refer patients with EoE to an allergist?
    1. Always
    2. More than 50% of the time
    3. Less than 50% of the time
    4. Never
    5. Only if they have a history of prior allergies
24. How often do you follow your EoE patients after initial diagnosis?
    1. Every 3 – 6 months
    2. Every 7 – 11.9 months
    3. Once yearly
    4. Once every 2 years
    5. No regular follow-up but will see them if they become symptomatic
25. How often will your patient with EoE have a repeat EGD performed after their initial diagnosis?
    1. All patients to evaluate for treatment effectiveness
    2. If they remain symptomatic
    3. If they have strictures requiring dilation
26. If you do perform a repeat EGD, how soon after initiating therapy would you consider repeating EGD?
    1. Less than 3 months
    2. 3 – 5.9 months
    3. 6 – 11.9 months
    4. At least 1 year later
    5. When they report symptoms recurrence
    6. Not applicable
27. How often do you specifically ask patients about adaptive behaviours (e.g., fear of aversive consequence from eating certain food, ARFID), when not voicing common dysphagia symptoms?
    1. Always
    2. Commonly (more than 50%)
    3. Occasionally (less than 50%)
    4. Never
28. Which guidelines do you follow in the assessment and management of EoE? (multi-select)
    1. American Gastroenterology Association (AGA) 2020
    2. American Society of Gastrointestinal Endoscopy (ASGE) 2022
    3. British Society of Gastroenterology (BSG) 2022
    4. None
29. Would you consider Canadian guidelines on the workup and management of EoE to be helpful in your practice?
    1. Yes
    2. No
